# Predictors of Workforce Retention Among Malawian Nurse Graduates of a Scholarship Program: A Mixed-Methods Study

**DOI:** 10.9745/GHSP-D-14-00170

**Published:** 2015-03-02

**Authors:** Kelly Schmiedeknecht, Melanie Perera, Ellen Schell, Joyce Jere, Elizabeth Geoffroy, Sally Rankin

**Affiliations:** a University of California, San Francisco, Global Health Sciences, San Francisco, CA, USA.; b Global AIDS Interfaith Alliance (GAIA), Limbe, Malawi.; c University of California, San Francisco, School of Nursing, San Francisco, CA, USA.

## Abstract

**Background:**

Malawi faces critical health care worker shortages of both physicians and nurses. The Global AIDS Interfaith Alliance (GAIA) began a nursing scholarship program in Malawi that requires graduates to work in the public sector for 4–5 years following graduation. The main objective of this study was to identify job satisfaction and retention factors of scholarship recipients after graduation.

**Methods:**

We conducted a mixed-methods study consisting of 30 individual qualitative interviews and 56 quantitative surveys that evaluated job satisfaction, factors associated with retention, and impact of the GAIA Nursing Scholarship Program. Participants included GAIA scholarship recipients who had graduated. We used thematic analysis to analyze qualitative interviews. Kruskal-Wallis, Spearman correlation, and chi-squared tests were used to analyze survey data.

**Results:**

The majority of survey and interview participants indicated it was unlikely that they would leave the public sector (70% and 90%, respectively). Most interview and survey participants cited a lack of supplies, inadequate human resources, and high workload as major challenges to their work. Poor working relationships with management or coworkers was significantly correlated with consideration of changing jobs in the next 6 months (correlation coefficient −0.28, *P* < .05 and −0.36, *P* < .01, respectively). Low salaries, high workload, poor accommodations, and a lack of appreciation were the most common reasons given for considering leaving the public sector while job security, desire to pursue further education, and public service agreement were primary motivations for continuing to work in the public system. Participants felt supported by GAIA staff and expressed a desire to serve their communities in return by working in government-supported health facilities.

**Conclusions:**

Despite the many challenges faced by public-sector nurses, low-income countries such as Malawi can employ non-remuneration strategies to retain nurses in the public sector, including adequate housing, availability of supplies, advancement opportunities, and positive work environments. Scholarship programs with close follow-up of graduates may also help increase retention.

## Abstract (precis)

Several non-remuneration strategies may help improve retention of public-sector nurses: availability of supplies, adequate housing, advancement opportunities, and a positive work environment. A scholarship program with close follow-up of graduates may also help improve retention.

## INTRODUCTION

Many countries in sub-Saharan Africa, including Malawi, face health care worker shortages, which directly affects the health of their populations. The estimated doctor-to-population ratio in Malawi is 0.2:10,000, and the nurse-to-population ratio is 3.4:10,000.^1^^–^^3^ The nursing ratio is one-third of the WHO's recommended 10 nurses for every 10,000 people, leaving Malawian health facilities with a 65% vacancy rate for nurses and nurse-midwives.^4^

Malawi's 3.4:10,000 nurse-to-population ratio is one-third of WHO's recommendation.

In 2004, the Malawi Ministry of Health (MOH) and the Christian Health Association of Malawi (CHAM) began a 6-year Emergency Human Resource Program (EHRP) to address the critical health worker shortage.^5^^,^^6^ The EHRP improved recruitment and retention of health workers in the country, expanded domestic health care training capacity, and employed international volunteer health care workers in the short term.^5^^,^^6^ Through this program, the overall population of nurses increased by 39% between 2004 and 2009.^5^ Despite the moderate success of the EHRP, the shortage of health workers in the public sector persists, with the largest vacancies in rural areas of Malawi.^5^

The MOH is the main supplier of health care, delivering about 76% of the country's services.^7^ CHAM also delivers essential services free of charge through a service-level agreement with the MOH, which provides funding for limited services and human resources for health.^6^^,^^8^ Although CHAM was established as a private health system made up of faith-based mission hospitals and training institutions, the MOH and CHAM work hand-in-hand to provide access to health care in rural areas.^6^^,^^8^ The MOH also deploys 40% of nurse graduates to CHAM facilities while the other 60% are deployed to MOH facilities.^9^

With such a large percentage of the population dependent on publicly funded health care, retention strategies must address not only workers leaving Malawi, but also internal brain drain and worker preference for the private sector and urban areas. Salary is thought to be a main reason why nurses in Malawi leave government and CHAM-run health facilities, but it is often not the only reason.^6^^,^^10^^,^^11^ Poor job satisfaction has been linked to burnout, absenteeism, intentions to leave, and high turnover rates.^12^

Low salaries as well as poor job satisfaction have been linked with high turnover in Malawi's public health care system.

One nonprofit organization attempting to address the nursing shortage in Malawi is the Global AIDS Interfaith Alliance (GAIA).^13^ GAIA offers preservice scholarships for nurses who need assistance with college fees and who demonstrate a commitment to work in the public health system after graduation. The scholarship program supports nursing students who are predominantly orphans or from lower socioeconomic backgrounds. This strategy aligns with the “transformative education agenda” recommendations from the Commission on Education of Health Professionals for the 21st Century.^14^ The scholarship recruitment phase targets needy students who have been accepted to nursing college (including one located in and serving a rural area) who are facing financial hardship and would otherwise be unable to complete a diploma/degree in nursing.^14^

The scholarship program aims to increase the number of nurses working in the public sector in 2 ways^13^:

- By providing tuition support, living stipends, uniforms, nursing supplies, and payment of council exam fees to improve yearly school progression of nursing students, on-time graduation rates, and licensing exam performance.
- By requiring students to sign a service agreement to work for the MOH or CHAM for 4–5 years after graduation from nursing school to retain them in the public sector.

The GAIA Nursing Scholarship Program provides tuition support to nursing students in Malawi and requires graduates to work for 4–5 years in the public health system in return.

GAIA monitors licensed GAIA nurse graduates deployed by the Malawi MOH into the public health system to ensure they fulfill their service agreement. While in school and after graduation (until 2 years after completion of the graduates' service agreement), GAIA maintains close follow-up with each scholar through site visits, text messaging, phone calls, and regional get-together events, providing academic assistance, psychosocial and clinical mentoring, and educational support, including opportunities for continuing professional development.^13^ GAIA staff maintain follow-up data on all graduates in a continually updated database. GAIA's follow-up activities assist the MOH and CHAM with tracking whether GAIA scholars have reported to deployment sites.

The program has been operating in Malawi in collaboration with the MOH, CHAM, and the training institutions since 2005 and has awarded more than 400 scholarships to nursing students at the technician, diploma, bachelor's, and master's level. Although the program started before the 2010 WHO policy recommendations to improve retention of health workers in remote and rural areas, we have implemented some of the WHO recommendations, including targeted admission policies, supporting students attending rural nursing colleges, designing and conducting continuing professional development events for GAIA scholars, and ensuring a compulsory service agreement after graduation.^15^

The objectives of this study were to understand predictors of workforce retention among Malawian nurse graduates from the GAIA scholarship program and evaluate the effectiveness of the scholarship program. Although not all CHAM employees are hired through the MOH, for the purpose of this paper we are considering those nurses working under the service agreement to be public employees and those working for NGOs or for-profit organizations to be private employees. Retention is defined as a graduate who is currently serving or has completed his/her service agreement at the facility assigned by the MOH or CHAM.

## METHODS

We undertook a mixed-methods study design consisting of semi-structured qualitative interviews and a survey. The study took place over a 6-week period between March 2014 and May 2014. The study was approved by the Committee on Human Research at the University of California San Francisco and the College of Medicine Research and Ethics Committee (COMREC) in Malawi. Prior to data collection, permission to conduct a program evaluation study was obtained from the MOH, CHAM, and GAIA. Written informed consent was obtained from all study participants.

**Figure:**
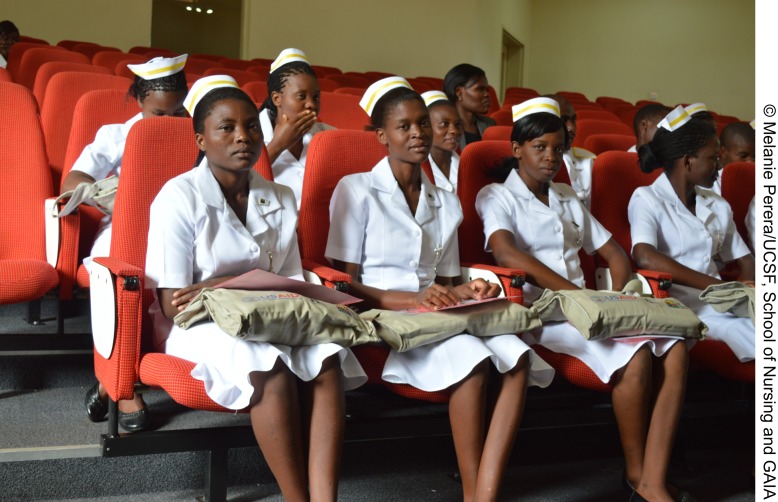
At their induction ceremony, GAIA scholarship recipients receive essential nursing supplies and uniforms.

### Selection of Participants

The survey was distributed to all scholarship recipients who had graduated and for whom GAIA had current contact information. The convenience sample for the qualitative interviews was selected from this same database of 156 graduated GAIA scholarship recipients. Inclusion criteria for the interviews included being in accessible districts during the study period (i.e., could be reached by phone, email, or in person), including those who were currently working within their service agreement time period, those who had completed their agreement, and those who had defaulted on their service agreement. Exclusion criteria included any graduate who had returned to school, was not deployed, or could not be reached by phone, email, or in person.

### Interview and Survey Instruments

Key interview topics were chosen based on a literature review of previous studies related to nurse retention, including job satisfaction factors such as preparedness, salary, availability of quality housing, availability of supplies, workload, opportunities for advancement, and relationships with coworkers and management.^16^^–^^23^ Additional questions specific to the GAIA Nursing Scholarship Program were asked to evaluate successes and identify areas for improvement. We used 2 questionnaires: one targeting patient care nurses and midwives and the other for nurse educators at colleges or universities.

The survey included the 20-item Satisfaction of Employees in Health Care (SEHC) instrument.^24^ This validated instrument measures health worker job satisfaction in low-income settings in areas of job content (condition of buildings, availability of supplies, workload, and organizational structure) and relationships with supervisors and coworkers.^24^ We also included questions about the GAIA Nursing Scholarship Program and the participants' future career plans.

### Data Collection

All interview and survey data were collected and analyzed by a single researcher who was not associated with GAIA staff.

We contacted eligible participants by phone for interviews, with a focus on reaching all 3 regions of Malawi and having variation among the type of facility and employer type and with consideration to urban-rural differences. The number of nurses ultimately interviewed in each region was directly proportional to the total number working in that region. Individual interviews took approximately 30–50 minutes to complete and were audio-recorded with permission of the participant.

Quantitative surveys were distributed in person to those who could be reached or by email. All graduates participating in the qualitative interviews also completed the quantitative survey before the interview. For those who had an email address on file and were unable to be reached in person, a web-based survey using Qualtrics was distributed. To ensure that graduates did not complete multiple surveys, the graduates were given unique identification numbers that they returned with the paper or web-based survey. The number assigned was destroyed following distribution to the graduates and was not associated with survey responses.

### Data Analysis

A single researcher analyzed the qualitative data using thematic analysis in Dedoose qualitative analysis software.^25^^,^^26^ Data were analyzed first by reading through transcripts to become familiar with the content. The researcher developed initial codes by highlighting key phrases and searching for themes among these codes. Through an iterative process, themes were further refined and categorized. Data were reduced by clustering topics and key ideas into a summary of impressions.^25^^,^^26^

Quantitative data were examined as part of a program evaluation and to enrich and support the themes uncovered in the qualitative analysis. Kruskal-Wallis, Spearman correlation, and chi-squared tests were used to analyze survey data as appropriate. For all tests, a *P* value of less than .05 was considered to be statistically significant.

## FINDINGS

Of the 156 GAIA scholarship recipients who had graduated, 141 met the inclusion criteria for the program evaluation study. We conducted 30 qualitative interviews throughout the 3 regions of Malawi in 10 districts (Figure). Surveys were sent to the GAIA scholars who had graduated and for whom we had current contact information; difficulties with Internet access and issues with the Qualtrics server resulted in a 36% response rate for the survey (N = 56).

**Figure.**
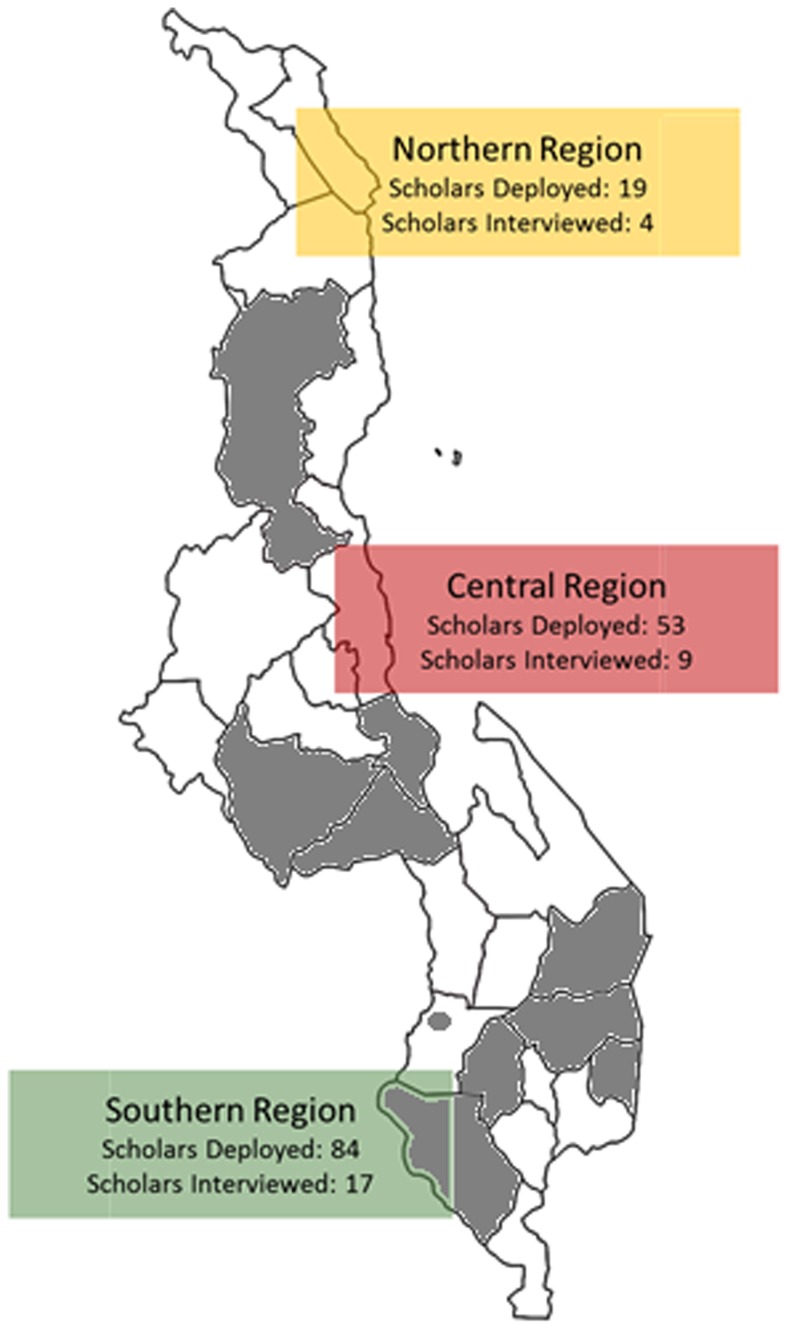
Locations of Deployed and Interviewed Global AIDS Interfaith Alliance (GAIA) Nursing Scholars Malawi consists of 3 separate administrative regions: North, Central, and South. Gray areas in the map represent districts where GAIA Scholars were interviewed.

The nurses interviewed were predominantly women, with 74% working in government facilities, 23% in CHAM facilities, and 3% for an NGO (survey participants had a similar employer profile) (Table 1). The mean age of interview participants was 30 years, with a range of 23 to 63 years. Interview participants had an average 5.6 years of nursing experience, with 2.8 years at their current facility. Most (80%) of the interview participants were currently working within their service agreement, 17% had completed their service agreement, and 1 participant (3%) had defaulted on her agreement. (Comparable data were not collected from survey participants.) Most of the interview participants were working in urban areas (67% urban, 23% rural, and 10% peri-urban).

**Table 1.** Demographic Characteristics of Interview and Survey Participants

****	**Interview Participants (N = 30)**	**Survey Participants^a^ (N = 56)**
Gender, n (%)		
Male	1 (3%)	1 (2%)
Female	29 (97%)	55 (98%)
Employer Type, n (%)		
Government	22 (74%)	42 (75%)
CHAM	7 (23%)	11 (20%)
NGO	1 (3%)	3 (5%)
Facility Type, n (%)		
Central hospital	10 (33%)	13 (23%)
District hospital	3 (10%)	15 (27%)
Health center	6 (20%)	7 (13%)
Mission hospital	3 (10%)	4 (7%)
Nursing college	6 (20%)	15 (27%)
Other	2 (7%)	2 (6%)

Abbreviation: CHAM, Christian Health Association of Malawi.

a All interview participants also participated in the survey.

Five major themes emerged from the qualitative interview data, which were also supported by the quantitative survey data (Table 2).

**Table 2.** Major Themes Derived From Qualitative Interviews With GAIA Nursing Scholars

Theme 1	Lack of resources and high nurse-to-patient ratios were main challenges faced by nurses.
Theme 2	Poor working relationship with management or other nurses were likely predictors of wanting to leave the current facility but not necessarily the public system.
Theme 3	Low salaries in relation to workload, poor housing options, and lack of appreciation were the most common reasons for leaving the public system.
Theme 4	Job security, continuing education, and public service agreement were primary motivations for continuing to work in the public system.
Theme 5	Nurses felt both supported and a sense of belonging, as well as a desire to give back to the community, due to the GAIA scholarship program.

Abbreviation: GAIA, Global AIDS Interfaith Alliance.

**Theme** **1: Lack of resources and high nurse-to-patient ratios were main challenges faced by nurses.** Participants cited a lack of resources, in terms of both supplies and staffing, and high nurse-to-patient ratios as reasons they were dissatisfied with their jobs. Almost all the interview participants (29/30) cited a lack of supplies as one of the greatest sources of dissatisfaction and the most challenging part of their job.

Lack of resources and high workload were the main challenges faced by nurses.

*With enough resources, I think the work would be easier. Because without resources, when we're improvising it's tiresome. You are using your head, you think too much. What can I do? What should I do? Everyone is looking at you while there is nothing you can do, nothing to use.* (Surgical nurse, MOH facility)

The only nurse who did not cite this as a reason was working for an NGO. Among survey respondents, 75% either disagreed or strongly disagreed that their department provided all the necessary resources and supplies to perform their duties (Table 3). Overall, this item received the lowest score among survey statements about sources of job satisfaction, with a mean rating of 2.2. There was no statistically significant difference between those working at CHAM and MOH facilities.

**Table 3.** Job Satisfaction Survey Findings, Malawi GAIA Nursing Scholarship Program Graduates

**Statement About Current Job**	**% Disagreed or Strongly Disagreed**	**% Agreed**
The management of this organization is supportive of me.	11%	89%
I receive the right amount of support and guidance from my direct supervisor.	20%	80%
I am provided with all trainings necessary for me to perform my job.	41%	59%
I have learned many new job skills in this position.	39%	61%
I feel encouraged by my supervisor to offer suggestions and improvements.	14%	86%
The management makes changes based on my suggestions and feedback.	41%	59%
I am appropriately recognized when I perform well at my regular work duties.	30%	70%
The organization rules make it easy for me to do a good job.	21%	79%
I am satisfied with my chances for promotion.	64%	36%
I have adequate opportunities to develop my professional skills.	39%	61%
I have an accurate written job description.	21%	79%
The amount of work I am expected to finish each week is reasonable.	48%	52%
My work assignments are always clearly explained to me.	23%	77%
My work is evaluated based on a fair system of performance standards.	46%	54%
My department provides all the equipment, supplies, and resources necessary for me to perform my duties.	75%	25%
My coworkers and I work well together.	7%	93%
I feel I can easily communicate with members from all levels of this organization.	11%	89%

Abbreviation: GAIA, Global AIDS Interfaith Alliance.

Interview participants expressed an emotional toll from being unable to provide appropriate care due to lack of resources. Inadequate staffing and high workload were also frequently identified as a challenge. One nurse explained:

*Like this season, malaria season, we normally have patients up to 300 against 8 nurses. We are supposed to be 8 nurses, but … you come here and find that there are only maybe 4 nurses and you work against 300 patients. It's a very busy ward and the job is just very stressful.* (Pediatric nurse, MOH facility)

**Theme 2: Poor working relationships with management or other nurses were likely predictors of wanting to leave the current facility but not necessarily the public system.** Interview participants stated the importance of feeling appreciated by their supervisors and emphasized positive relationships with coworkers as factors associated with remaining in their current job. Three participants interviewed had previously transferred, and another was planning a transfer in the future because of a poor relationship with coworkers or management. Some participants felt that management in the private sector was generally harder on staff, less understanding of their personal lives, and more interested in making money than those in the public sector.

Nurses emphasized the importance of feeling appreciated by their supervisors.

A nurse in a rural health center indicated that her relationship with management and her coworkers was a reason for her wanting to remain at her work site:

*What I like most is that mostly the workers here are able to communicate effectively. We are united. We understand each other well. It's easy for us to cooperate and achieve one goal. That's what I like most.* (Health center nurse, CHAM facility)

One nurse who requested to be transferred to a different health facility in the CHAM system explained:

*The management team has more effect on whether a nurse can be at a facility for a long time. It depends on the treatment you are receiving. If you are treated well you can … be there forever, but if you are not treated well it is easy for you to look for another place to work.* (Outpatient nurse, CHAM facility)

Similarly, the surveys found that higher scores on the relationship with management scale (−0.28, *P* <.05), as well as higher scores on the relationship with coworkers scale (−0.36, *P* < .01), were negatively correlated with how likely a participant was to be interested in looking for a new job in the next 6 months (Table 3). Higher scores on the relationship with management scale were also negatively correlated with how likely a participant was to be interested in looking for a new job within the public sector (−0.33, *P* = .02) (Table 4). The survey data showed no other items to be significantly correlated with seeking a new job in the public health sector. There was also no statistically significant difference between those working for the MOH vs. CHAM.

**Table 4.** Spearman Correlations Between Job Satisfaction Measures and Likelihood of Leaving the Public Sector

**Likelihood of Leaving Current Public-Sector Position to:**	**Job Satisfaction Measures**			
	**Relationship With Management**	**Job Content**	**Relationship With Coworkers**	**Total**
Look for a new job in the next 6 months	**−0.28 (.04)**	−0.21 (.14)	**−0.36 (.01)**	**−0.30 (.03)**
Look for a new job in the next year	−0.20 (.15)	−0.13 (.34)	**−0.32 (.02)**	−0.23 (.09)
Look for a new job after service agreement is completed	−0.23 (.11)	−0.03 (.87)	−0.02 (.94)	−0.16 (.24)
Look for a different job in the public health system	**−0.33 (.02)**	−0.19 (.17)	−0.16 (.23)	**−0.30 (.03)**
Look for a job with a private employer	−0.15 (.26)	0.02 (.92)	0.05 (.74)	−0.10 (.47)
Look for a job outside the health care profession	−0.11 (.42)	0.00 (.99)	0.00 (.99)	−0.06 (.68)
Continue formal education	0.03 (.80)	0.15 (.30)	0.00 (.98)	0.07 (.61)
Look for a job outside Malawi	0.01 (.94)	0.14 (.33)	0.12 (.40)	0.09 (.51)

All data are presented as the correlation coefficient (*P* value). Negative correlation coefficients represent an inverse relationship while positive coefficients represent a constant relationship.

Data shown in boldface are statistically significant at *P* < .05.

**Theme 3: Low salaries in relation to workload, poor housing options, and lack of appreciation were the most common reasons for leaving the public system.** Although interview participants felt that resources were a major contributor to nurses' job dissatisfaction, low salary was mentioned even more frequently as a reason nurses were leaving MOH or CHAM facilities.

*The issue is about cash. [The private sector] is paying their nurses much money as compared to what they are receiving in CHAM; they do talk of leaving CHAM and joining some other facilities.* (Maternity nurse, CHAM facility)

Among the 5 nurses interviewed who had completed their service agreement, 4 have remained in MOH or CHAM facilities. The one who left the public system was currently employed privately and cited an increase in salary as her primary motivation for seeking new employment.

Other reasons participants cited for leaving the public sector were high workload, poor accommodations, and lack of appreciation.

*I cannot manage to work the rest of my life in the public hospitals … because there is too much work overload.* (District hospital nurse, MOH facility)

Participants also reported a lack of appreciation for nurses by the general public.

*No, in Malawi people don't value nursing … they just take us for granted. It's a demotivation because you are not appreciated. I think that's why some people always think of going to other places to work there, where they can be appreciated. Another country or maybe another organization.* (Male ward nurse, MOH facility)

A few participants mentioned that housing accommodations were not immediately available, which delayed deployment or required reassignment. Because of this delay, some nurses needed to look for their own employment outside the government before being deployed:

*The government didn't deploy us [right away], so I had to search for somewhere to work for my own. So I got employed [privately].* (Health center nurse, NGO)

Housing accommodations were not always immediately available after graduation.

The transition between school and deployment was a difficult time for many of the participants. Most came from poor families that could not offer assistance when the participants could not afford to pay for their own housing. Several of the nurses took private jobs prior to their deployment by the MOH to supplement their income.

Survey data showed no statistical significance between any of the job satisfaction scales and likeliness to leave the public sector (Table 4). The majority of survey participants (70%) indicated that it was unlikely that they would leave the MOH or CHAM facilities.

The majority of surveyed nurses said it was unlikely they would leave the public sector.

**Theme 4: Job security, continuing education, and public service agreement were primary motivations for continuing to work in the public system.**

Nearly all the nurses interviewed (27/30) stated that they did not currently have plans to leave the public system now or after their service agreement was completed. There were no differences in plans to leave between those working at CHAM vs. MOH facilities in either the interviews or the surveys.

All participants expressed a desire to further their education through training and to pursue further education. Although they admitted such opportunities were rare, the belief that they might have the chance to continue their education motivated them to stay in the public sector and serve their communities. The 4 interview participants who completed their service agreement and remained in the public system all stated that they hoped to have the opportunity to continue their education.

In addition to continuing education opportunities, the other most common reason cited for continuing in government and CHAM facilities was job security. One nurse discussed how there is more freedom and security within the government system:

*Most of the nurses like working in the public sector because it's free—what can I say? The rules are there, but they're not very stressed like in private hospitals … you can say you are sick and won't make it to work and they will understand. But when you are in private sector, it's very rare that you can do that. Mostly I feel like the benefits are the freedom in the public sector and that's what makes people stay.* (Health center nurse, NGO)

Several nurses mentioned the desire to work in the public system because it is where they can best serve their communities. One nurse who has completed her service agreement explained why she has chosen to stay at her current job:

*I prefer the public system because I feel that is where I can benefit the most [people]. When I went for my training my aim was to work where I could serve the Malawian people at large. Because I know that most of the people that are affected by these conditions or illnesses in Malawi are the poor people. So where can you meet the poor people? These people, you meet them in the public institutions. So I feel better that if I work in the public institutions I can serve the Malawians at large unlike if I go to a private sector.* (Health center nurse, MOH facility)

**Theme** **5: Nurses felt both supported and a sense of belonging, as well as a desire to give back to the community, due to the GAIA scholarship program.** Interview participants felt supported by GAIA staff and indicated a sense of belonging through the program. Before graduation, the GAIA program elements receiving the highest ratings from survey participants (average score 4.0 or higher) were related to the monetary benefits (payment of fees, monthly stipend, clinical supplies, uniforms and shoes) (Table 5). After graduation, the highest score was for check-ins with GAIA staff (mean = 4.5).

**Table 5.** Value Ratings of the GAIA Nursing Scholarship Program (1–5 Scale)

**Program Aspects**	**n**	**Mean**	**SD**	**Median**
Before Graduation				
Payment of fees	52	4.9	0.5	5.0
Monthly stipend	48	4.2	1.2	5.0
Get-togethers	49	3.8	1.3	4.0
Clinical supplies	47	4.0	1.2	5.0
Uniforms and shoes	46	4.0	1.3	4.0
Talks by role models	46	3.7	1.4	4.0
Check-ins with GAIA staff	49	3.7	1.5	4.0
Availability of GAIA staff for assistance	51	3.6	1.5	4.0
After Graduation				
Get-togethers	48	3.7	1.5	4.0
Talks by role models	44	3.4	1.5	3.5
Check-ins with GAIA staff	52	4.0	1.2	4.5
Site visits with staff/donors	46	3.7	1.5	4.0
Availability of GAIA staff for assistance	50	3.5	1.4	4.0

Abbreviation: GAIA, Global AIDS Interfaith Alliance; SD, standard deviation.

Scholarship graduates rated very highly the regular check-ins with scholarship program staff post-graduation.

Interview participants expressed a desire to give back to the community because they were given the scholarship opportunity. They also reported that their relationships with the GAIA staff and other scholars extended beyond the element of monetary support.

*It's good because it's not only that you have paid fees for me but it's also like a friendship. We have started a friendship and we can build from that. Not just a monetary bond, but also a friendship bond.* (Central hospital nurse, MOH facility)

Several participants mentioned GAIA staff supported them through problematic situations. For example, GAIA staff helped one participant navigate a difficult deployment through CHAM:

*I remember when I talked to her [GAIA staff] about what happened at my institution she was like, “Oh, I never knew about this.” So for me, I felt good that she was able to help. She went to CHAM and discussed it.* (Nurse, CHAM facility)

Many participants mentioned the importance of close follow-up by GAIA staff. A participant working as a clinical instructor believed follow-up by GAIA staff has helped retain nurses in the public sector where the MOH has failed:

*If I'm faithful and I am committed, they [GAIA] will be able to sponsor other Malawians and help increase the health care in the public sector. With what GAIA is doing they are able to keep all of us in the public sector. But [the MOH], I think it is giving out to the community, paying fees for nurses and they are running away simply because the [nurses] don't have proper channels to follow. Because they don't keep in touch with them.* (Nurse educator, CHAM facility)

**Figure:**
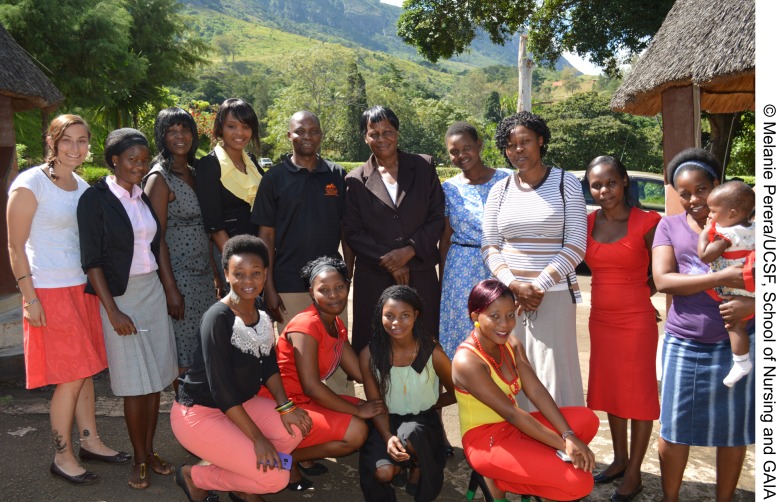
GAIA scholars working in 3 districts of Malawi attend a regional get-together to follow-up with the program after graduation and deployment and for professional networking opportunities.

## DISCUSSION

Although there is abundant research documenting health care worker shortages and brain drain in sub-Saharan Africa, there is little that connects retention factors with programs that can motivate and retain nurses.^11^^,^^12^^,^^27^^–^^29^ This evaluation was conducted to examine retention factors among graduates of the GAIA Nursing Scholarship Program and to inform future programming.

The biggest challenges and sources of job dissatisfaction for Malawian nurses in this study were low pay, lack of resources, and staffing shortages. Most of the nurses did not indicate these as reasons to consider requesting a different deployment site through the MOH or CHAM, but lack of resources was mentioned as a reason to consider leaving the public system for private NGOs. As in many other countries, salary, perceived to be better within the private sector, appears to be the most important factor in prompting a move from the public to the private sector.^21^^,^^27^^,^^29^^–^^34^

Major sources of job dissatisfaction for nurses were low pay, lack of resources, and staffing shortages.

Although most of the participants denied a personal desire to leave the public health sector, all expressed that Malawian nurses, in general, would rather work in the private sector. For low-income countries such as Malawi, matching the levels of remuneration provided by the private sector is not currently a feasible option.^32^ Other studies have suggested that non-salary retention strategies are associated with increased job satisfaction and retention. These include incorporating opportunities for education and advancement, ensuring adequate accommodations near the hospital, reducing workload, and creating a work environment that encourages collaboration among staff and appreciation from management.^7^^,^^27^^,^^29^^–^^34^

Non-remuneration strategies to increase job satisfaction and retention include advancement opportunities, housing accommodations, and a positive work environment.

A recurring theme in this study was a desire to have open communication with management and to feel appreciated for their work. The interviewed nurses suggested that there were not many channels to be recognized or appreciated for their hard work, and that supervisors were instead quick to reprimand them. They believed this attitude and treatment begins with their low salaries. Developing channels for communication and constructive feedback with supervisors could help nurses to feel valued and appreciated.^29^

All nurses expressed a desire to further their education through training workshops and advanced degrees. Although they acknowledged that such opportunities were rare in the public system, the chance for professional advancement motivated them to stay and serve their communities in the hopes that they would be rewarded. A study in Tanzania also found that medical doctors preferred the public sector for the opportunity to pursue additional education.^30^

Although increasing the number of nurses is a critical piece to solving the human resource crisis, retaining well-educated nurses where they are needed most is equally important. Focusing on non-wage-based strategies could motivate nurses to remain in the public sector. Ensuring adequate housing, especially for those being deployed to rural areas, is one way to increase satisfaction among nurses. In our study, nurses in rural areas were unsatisfied with accommodations and felt that this was a major area for the MOH to improve job satisfaction. Delays in deployment and lack of housing can result in prolonged staffing shortages and financial hardship for nurse graduates. This corresponds with results found in a Tanzanian study of medical doctors and dental surgeons, which found similar issues, naming the problem the “lost in transition syndrome.”^28^

The GAIA Nursing Scholarship Program was designed to give qualified young Malawians, who could otherwise not afford school, the opportunity to pursue an education in nursing. The close follow-up and relationship between GAIA staff and graduates resulted in 97% of the GAIA scholars remaining in the public sector throughout their service agreement and the majority continuing to at least 2 years past their service agreements.^35^ This is supported by other research that found limited success with solely bonding graduates to the public sector due to issues of deployment and follow-up.^.^^36^^,^^37^ Nurses in this study believed that they should give back to their communities because of the opportunity that was given to them through GAIA and felt that the supportive relationships with GAIA staff and other GAIA scholars helped them transition into the workforce.

Scholarship programs with close follow-up of graduates may help improve retention in the public sector.

### Limitations

Because participants were deployed nationwide, it was not possible to randomize the sample for the interviews or in-person surveys. There were a limited number of participants who completed their service agreement, and we did not interview or survey nurses who were not part of the GAIA scholarship program. Those that completed the online survey were more likely to be those in urban areas where access to the Internet was better than in some rural areas. Although we ensured that the interviewer was not associated with GAIA staff, it is still likely that there is some response bias. Despite these limitations, the study has practical applications for improving job satisfaction and retention among nurses working in low-resource settings. Additionally, lessons can be learned from the success of GAIA's Nursing Scholarship Program in retaining nurses in the public sector in Malawi.

## CONCLUSIONS

Nurses working in Malawi's public health system face several challenges, including lack of supplies, shortage of human resources, limited housing options in rural areas, and low pay. Despite these challenges, nurses report many reasons to remain working in the public sector, such as opportunities for advancement, positive relationship with coworkers, and constructive feedback from managers. In addition to non-remuneration strategies, programs such as the GAIA Nursing Scholarship Program can help increase retention of health care workers by creating loyalty through careful follow-up monitoring both during and after schooling.

## Notes

**Peer Reviewed**

## References

[1] World Health Organization (WHO). Malawi: health profile. Geneva: WHO; 2014 Available from: http://www.who.int/gho/countries/mwi.pdf?ua = 1

[2] World DataBank [Internet]. Washington (DC): World Bank Group; Malawi: World development indicators. 2005 - [cited 2014 Jul 14]. Available from: http://databank.worldbank.org/data/views/reports/tableview.aspx

[3] Republic of Malawi; Health Metrics Network. Health information systems assessment report. Lilongwe (Malawi): Ministry of Health [Malawi]; 2009 Available from: http://www.who.int/healthmetrics/library/countries/2HMN_MWI_Assess_Final_2009_07_en.pdf

[4] United States President's Emergency Plan for AIDS Relief (PEPFAR) [Internet]. Washington (DC): PEPFAR; c2011 Nursing Education Partnership Initiative (NEPI) in Malawi; [date unknown] [cited 2015 Feb 7]; [about 1 screen]. Available from: http://www.pepfar.gov/partnerships/initiatives/nepi/programs/malawi/index.htm

[5] Management Sciences for Health (MSH); Management Solutions Consulting (MSC). Evaluation of Malawi's Emergency Human Resource Programme. Cambridge (MA); MSH; 2010 Available from: http://www.who.int/workforcealliance/media/news/2010/Malawi_MSH_MSC_EHRP_Final.pdf

[6] Global Health Workforce Alliance (GHWA) Task Force on Scaling Up Education and Training for Health Workers. Country case study: Malawi's Emergency Human Resources Programme. Geneva: World Health Organization, GHWA; 2011 Available from: http://www.who.int/workforcealliance/knowledge/case_studies/CS_Malawi_web_En.pdf?ua = 1

[7] Grigulis AI Prost A Osrin D. The lives of Malawian nurses: the stories behind the statistics. Trans R Soc Trop Med Hyg. 2009;103(12): 1195–1196. 10.1016/j.trstmh.2009.03.005. 19349055 PMC3428889

[8] Chirwa ML Kazanga I Faedo G Thomas S. Promoting universal financial protection: contracting faith-based health facilities to expand access – lessons learned from Malawi. Health Res Policy Syst. 2013;11(1): 27. 10.1186/1478-4505-11-27. 23958156 PMC3751183

[9] Ministry of Health (MOH) [Malawi]. Nurse/midwife training operational plan: field assessments, analysis and scale-up plans for nurse training institutions. Lilongwe (Malawi): MOH; 2011 Available from: https://www.k4health.org/sites/default/files/Malawi_National%20Nurse%20Training%20Operational%20Plan.pdf

[10] Mangham LJ Hanson K. Employment preferences of public sector nurses in Malawi: results from a discrete choice experiment. Trop Med Int Health. 2008;13(12): 1433–1441. 10.1111/j.1365-3156.2008.02167.x. 18983274

[11] Mangham L Addressing the human resource crisis in Malawi's health sector: employment preferences of public sector registered nurses. London: Overseas Development Institute; 2007 Available from: http://www.odi.org/sites/odi.org.uk/files/odi-assets/publications-opinion-files/2505.pdf

[12] Blaauw D Ditlopo P Maseko F Chirwa M Mwisongo A Bidwell P. Comparing the job satisfaction and intention to leave of different categories of health workers in Tanzania, Malawi, and South Africa. Glob Health Action. 2013;6:19287. 10.3402/gha.v6i0.19287. 23364090 PMC3556679

[13] Global AIDS Interfaith Alliance. Nursing Scholarship Fund: Global AIDS Interfaith Alliance [Internet]. Larkspur, (CA): GAIA 2013 Available from: http://www.thegaia.org/

[14] Frenk J Chen L Bhutta ZA Cohen J Crips N Evans T. Health professionals for a new century: transforming education to strengthen health systems in an interdependent world. Lancet. 2010;376(9756): 1923–1958. 10.1016/S0140-6736(10)61854-5. 21112623

[15] World Health Organization (WHO). Increasing access to health workers in remote and rural areas through improved retention. Geneva: WHO; 2010 http://www.searo.who.int/nepal/mediacentre/2010_increasing_access_to_health_workers_in_remote_and_rural_areas.pdf 23741785

[16] Bradley S McAuliffe E. Mid-level providers in emergency obstetric and newborn health care: factors affecting their performance and retention within the Malawian health system. Hum Resour Health. 2009;7:14. 10.1186/1478-4491-7-14. 19228409 PMC2657772

[17] Siyam A Dal Poz M R, editors. Migration of health workers: WHO code of practice and the global economic crisis. Geneva: World Health Organization; 2014 Available from: http://www.who.int/hrh/migration/14075_MigrationofHealth_Workers.pdf

[18] Mackey TK Liang BA. Restructuring brain drain; strengthening governance and financing for health worker migration. Glob Health Action. 2013;6:1–7. 10.3402/gha.v6i0.19923. 23336617 PMC3547121

[19] Manafa O McAuliffe E Maseko F Bowie C MacLachlan M Normand C. Retention of health workers in Malawi: perspectives of health workers and district management. Hum Resour Health. 2009;7:65. 10.1186/1478-4491-7-65. 19638222 PMC2722569

[20] Beaglehole R Dal Poz MR. Public health workforce: challenges and policy issues. Hum Resour Health. 2003;1(1): 4. 10.1186/1478-4491-1-4. 12904251 PMC179882

[21] Brugha R Kadzandira J Simbaya J Dicker P Mwapasa V Walsh A. Health workforce responses to global health initiatives funding: a comparison of Malawi and Zambia. Hum Resour Health. 2010;8(1): 19. 10.1186/1478-4491-8-19. 20701749 PMC2925328

[22] Araújo E Maeda A How to recruit and retain health workers in rural and remote areas in developing countries: a guidance note. Washington (DC): World Bank; 2013 Available from: http://www-wds.worldbank.org/external/default/WDSContentServer/WDSP/IB/2013/06/17/000445729_20130617151929/Rendered/PDF/785060WP0HRHDC00Box377346B00PUBLIC0.pdf

[23] Coetzee S Klopper H Ellis S Aiken LH. A tale of two systems–nurses practice environment, well being, perceived quality of care and patient safety in private and public hospitals in South Africa: a questionnaire survey. Int J Nurs Stud. 2013;50(2): 162–173. 10.1016/j.ijnurstu.2012.11.002. 23218020

[24] Alpern R Canavan ME Thompson JT McNatt Z Tatek D Lindfield T. Development of a brief instrument for assessing healthcare employee satisfaction in a low-income setting. PLoS One. 2013;8(11): e79053. 10.1371/journal.pone.0079053. 24223878 PMC3818514

[25] Sandelowski M. Qualitative analysis: what it is and how to begin. Res Nurs Health. 1995;18(4): 371–375. 10.1002/nur.4770180411. 7624531

[26] Braun V Clarke V Using thematic analysis in psychology. Qual Res Psych. 2006;3(2): 77–101 10.1191/1478088706qp063oa

[27] Poppe A Jirovsky E Blacklock C. Why sub-Saharan African health workers migrate to European countries that do not actively recruit: a qualitative study post-migration. Glob Health Action. 2014;7:24071. 10.3402/gha.v7.24071. 24836444 PMC4021817

[28] Sirili N Kiwara A Nyongole O Frumence G Semakafu A Hurtig AK Addressing the human resource for health crisis in Tanzania: the lost in transition syndrome. Tanzan J Health Res. 2014;16(2): 1–9 10.4314/thrb.v16i2.6 26875304

[29] Iwu CG Rethinking issues of migration and brain drain of health-related professionals: new perspectives. Med J Soc Sci. 2014;5(10): 198–204 10.5901/mjss.2014.v5n10p198

[30] Tabatabai P Prytherch H Baumgarten I Kisanga OME Schmidt-Ehry B Marx M. The internal migration between public and faith-based health providers: a cross-sectional, retrospective and multicentre study from southern Tanzania. Trop Med Int Health. 2013;18(7): 887–897. 10.1111/tmi.12107. 23914366

[31] Pillay R. Work satisfaction of professional nurses in South Africa: a comparative analysis of the public and private sectors. Hum Resour Health. 2009;7(1): 15. 10.1186/1478-4491-7-15. 19232120 PMC2650673

[32] Cometto G Tulenko K Muula AS Krech R. Health workforce brain drain: from denouncing the challenge to solving the problem. PLoS Med. 2013;10(9): e1001514. 10.1371/journal.pmed.1001514. 24068895 PMC3775719

[33] Palmer D. Tackling Malawi's human resources crisis. Reprod Health Matters. 2006;14(27): 27–39. 10.1016/S0968-8080(06)27244-6. 16713877

[34] George G Atujuna M Gow J. Migration of South African health workers: the extent to which financial considerations influence internal flows and external movements. BMC Health Serv Res. 2013;13(1): 297. 10.1186/1472-6963-13-297. 23919539 PMC3765273

[35] Schell E Geoffroy E Perera M Jere J Laviwa J Schafer T Keeping nurses working where they are needed most: workforce retention results of a scholarship program in Malawi. Presented at: 20th Annual International AIDS Conference; 2014 Jul 20–25; Melbourne, Australia. E-poster available from: http://pag.aids2014.org/EPosterHandler.axd?aid = 6120

[36] Zimbudzi E Stemming the impact of health professional brain drain from Africa: a systemic review of policy options. J Public Health Africa. 2013;4(1): e4 10.4081/jphia.2013.e4 PMC5345423 28299093

[37] Eastwood JB Conroy RE Naicker S West PA Tutt RC Plange-Rhule J. Loss of health professionals from sub-Saharan Africa: the pivotal role of the UK. Lancet. 2005;365(9474): 1893–1900. 10.1016/S0140-6736(05)66623-8. 15924988

